# Entorhinal Subfield Vulnerability to Neurofibrillary Tangles in Aging and the Preclinical Stage of Alzheimer’s Disease

**DOI:** 10.3233/JAD-215567

**Published:** 2022-05-31

**Authors:** Josué Llamas-Rodríguez, Jan Oltmer, Douglas N. Greve, Emily Williams, Natalya Slepneva, Ruopeng Wang, Samantha Champion, Melanie Lang-Orsini, Bruce Fischl, Matthew P. Frosch, André J.W. van der Kouwe, Jean C. Augustinack

**Affiliations:** a Department of Radiology, Athinoula A. Martinos Center for Biomedical Imaging, Massachusetts General Hospital, Charlestown, MA, USA; b Department of Neuropathology, Massachusetts General Hospital, Boston, MA, USA; c CSAIL/HST, MIT, Cambridge, MA, USA

**Keywords:** Aging, cross-sectional, entorhinal cortex, histological labeling, immunohistochemistry, neuropathology, tauopathy, temporal lobe, three-dimensional imaging, validation study

## Abstract

**Background:**

Neurofibrillary tangle (NFT) accumulation in the entorhinal cortex (EC) precedes the transformation from cognitive controls to mild cognitive impairment and Alzheimer’s disease (AD). While tauopathy has been described in the EC before, the order and degree to which the individual subfields within the EC are engulfed by NFTs in aging and the preclinical AD stage is unknown.

**Objective:**

We aimed to investigate substructures within the EC to map the populations of cortical neurons most vulnerable to tau pathology in aging and the preclinical AD stage.

**Methods:**

We characterized phosphorylated tau (CP13) in 10 cases at eight well-defined anterior-posterior levels and assessed NFT density *within* the eight entorhinal subfields (described by Insausti and colleagues) at the preclinical stages of AD. We validated with immunohistochemistry and labeled the NFT density ratings on *ex vivo* MRIs. We measured subfield cortical thickness and reconstructed the labels as three-dimensional isosurfaces, resulting in anatomically comprehensive, histopathologically validated tau “heat maps.”

**Results:**

We found the lateral EC subfields ELc, ECL, and ECs (lateral portion) to have the highest tau density in semi-quantitative scores and quantitative measurements. We observed significant stepwise higher tau from anterior to posterior levels (*p* < 0.001). We report an age-dependent anatomically-specific vulnerability, with all cases showing posterior tau pathology, yet older individuals displaying an additional anterior tau burden. Finally, cortical thickness of each subfield negatively correlated with respective tau scores (*p* < 0.05).

**Conclusion:**

Our findings indicate that posterior-lateral subfields within the EC are the most vulnerable to early NFTs and atrophy in aging and preclinical AD.

## INTRODUCTION

The entorhinal cortex (EC) functions as the main gateway for cortical information to the hippocampus [1]. Abnormally hyperphosphorylated and misfolded tau protein accumulates in neurons to form neurofibrillary tangles (NFTs), which distribute in a predictable and hierarchical pattern in the first stages of AD [2–8]. NFTs develop early in the EC, making it essential to understanding Alzheimer’s disease (AD) [4, 5]. It is known that neurons in layer II of the EC are particularly vulnerable to age-related pathology [4, 5, 9–11]. In agreement with pathological reports, investigations of atrophy patterns have revealed alterations at the preclinical stage of AD, highlighting the predictive potential of early AD tracking in this region [12–14].

Some advances have been made using *in vivo* and *ex vivo* neuroimaging approaches in understanding the substructure of the EC. A high resolution functional magnetic resonance imaging (fMRI) report identified functional subdivisions and connectivity between the perirhinal cortex and the anterior-lateral EC, and between the parahippocampal cortex and the posterior-medial EC in the human brain [15]. Another fMRI study has demonstrated that lateral neurons in the EC are vulnerable to metabolic deficits in preclinical AD cases [16]. In addition, Leng and colleagues identified the transcription factor RORB as a molecular marker of neurons susceptible to NFTs in the caudal EC [17]. Animal studies have shown the functional relevance of medial temporal lobe areas and the connectivity to other cortical regions [1, 18–27], while studies in the human brain have singled out homologous regions using fMRI [1, 15, 28]. Anatomically, Insausti and colleagues subdivided the human EC into eight subfields based on distinct cytoarchitecture: EO (olfactory), ER (rostral), EMI (medial intermediate), EI (intermediate), ELr (lateral rostral), ELc (lateral caudal), ECs (caudal), and ECL (caudal limiting) [29]. Still, entorhinal sub-functions, pathologic vulnerability, and/or cognitive resilience remain an open and vital question in the human brain.

Pathologic diagnosis relies on two-dimensional data, and as such, the bulk of AD histology studies have been limited to just a few sections in the medial temporal lobe. Thus, the three-dimensional axes have been vastly understudied regarding NFT formation and have lacked subfield specificity, which could pose functional implications for early disease stages. Initial *ex vivo* MRI investigations have undertaken three-dimensional mappings of NFTs in the medial temporal lobe regions including the entorhinal, hippocampus, amygdala, and temporal pole areas [30–33]. Based on these initial scores, subsequent mappings have been combined to construct a 3D probabilistic atlas of medial temporal lobe (MTL) pathology [31]. Previous *ex vivo* reports, however, have lacked entorhinal subdivision specificity and histopathology validation approaches. And, of course, *in vivo* studies fail to provide this specificity as well due to poor spatial resolution and lack of ground truth tissue samples.

To address these issues, we characterized phosphorylated tau and specifically assessed NFT density *within* the EC subfields and at well-defined anterior-posterior anatomical levels. We focused exclusively on the preclinical stages of AD, the first two Braak and Braak stages [5]. This characterization yields a three-dimensional map of phosphorylated tau (CP13) vulnerability throughout the EC in 10 human brains at the earliest AD stages. With these scores, we determined anatomically-specific and age-dependent deposition of tau burden along the anterior-posterior axis. We present histopathologically-validated tau density “heat maps” on *ex vivo* MRIs with corresponding cortical thickness measures on all cases. The combination of methods—*ex vivo* MRI and histology—provide validation and allow for application to neuroimaging and will be useful for future *in vivo* imaging hypotheses. These descriptive maps bring together at least three factors of vulnerability: age, anatomical location, and phosphorylated tau immunoreactivity, showing the entorhinal tau vulnerability in 3D at the preclinical stages.

## METHODS

### Brain samples

We studied 10 human brain hemispheres (*n* = 3 right, *n* = 7 left) received from the Department of Neuropathology, Autopsy Suite at Massachusetts General Hospital (Boston, MA). All samples were collected in accordance with the internal review board of Massachusetts General Hospital. All experiments met ethical standards and were approved by the internal review board of Massachusetts General Hospital. The samples comprised five males, four females, and one case with demographic information unavailable. Ages ranged from 59–84 years, 69.67±9.94; mean±std (Table 1). Brain hemispheres were classified in this study as youngest-old (ages 59–74) or middle-old (ages 75–84) as defined by previous studies [34, 35]. We did not have any cases in the oldest-old category (ages 85 and above). All cases had a postmortem interval less than 24 h, and brain weights ranged from 1030–1402 g (1251.77±121.55; mean±std). All cases were fixed in 10% formalin and stored in 2% periodate-lysine-paraformaldehyde solution during MRI scanning.

**Table 1:** Demographic information for 10 brains studied. All cases were neurologic controls with no clinical symptoms

Case	Hemi	Age	Sex	Dx	Braak Stage	Aβ	TDP-43	*α*-Syn	PMI (h)	BW (g)	Cause of Death
1	LH	59	M	NC	I	neg	severe	neg	20	1,319	Surgery Complication
2	LH	59	F	NC	II	pos	moderate	neg	< 24	1,402	Alveolar Damage
3	RH	60	M	NC	II	neg	severe	neg	< 24	1,166	Liver Failure
4	RH	60	M	NC	I	neg	severe	neg	14	1,414	Aortic Dissection
5	LH	70	F	NC	I	pos	severe	neg	23	1,030	Myocardial Infarction
6	LH	n/a	n/a	NC	II	neg	moderate	neg	< 24	n/a	n/a
7	LH	73	F	NC	II	neg	moderate	neg	< 24	1,142	Visceral Hemorrhage
8	RH	80	M	NC	II	pos	severe	neg	< 24	1,348	Aortic Dissection
9	LH	82	M	NC	II	pos	severe	neg	< 24	1,224	n/a
10	LH	84	F	NC	II	pos	low	neg	< 24	1,221	Pneumonia

AD, Alzheimer’s disease; Aβ, amyloid-β; *α*-Syn, alpha-synuclein; BW, brain weight; Dx, clinical diagnosis; Hemi, Hemisphere; LH, left hemisphere; NC, cognitive control; n/a, not available; neg, negative; PMI, postmortem interval; RH, right hemisphere, TDP, Tar DNA-Binding Protein.

### Controlling for comorbidities

Based on clinical history, none of the cases had a diagnosis of Alzheimer’s disease (AD) nor any other neurological diseases. We followed the NIA Alzheimer’s Association guidance [36] to control for potential co-incidental mixed pathologies in AD. All cases were screened by the Massachusetts General Hospital Autopsy Suite (MPF, SC, MLO) for infectious disease (i.e., HIV, prion diseases, hepatitis, and COVID-19) and suspected infectious cases were excluded from the study. Cases were also excluded if they exhibited vascular deficits: cerebrovascular strokes, discolored white matter or lateral ventricle dilation (gross tissue inspection), and lacunar infarcts (Luxol fast blue/H&E stain). All cases showed a dark substantia nigra and were negative for *α*-synuclein, confirming that cases were negative for Parkinson’s disease. The tissue was also immunostained for Tar DNA-Binding Protein (TDP-43), a significant neurodegenerative marker [37, 38] that implies comorbidity with AD pathology [39, 40]. All demographic and diagnostic details are listed in Table 1.

### Ex vivo MRI acquisition

Cases were scanned in a whole body high-field 7T Siemens Magnetom (Siemens Healthineers, Erlangen, Germany). Two radio frequency coils were used: a 4-turn solenoid coil, giving a resolution of 100*μ*m isotropic providing full Fourier encoding [41], and the second, a 31-channel phased array coil with birdcage with an average isotropic resolution of 109*μ*m [42]. Medial temporal lobe tissue was blocked from the hemisphere, packed into a 50 ml test tube, and scanned in 2% periodate-lysine-paraformaldehyde or Fomblin (Solvay USA Inc., Princeton, NJ). A fast-low-angle-shot (FLASH) sequence with 3D encoding was used with flip angle 25° to yield optimal contrast in *ex vivo* tissue to visualize and distinguish microanatomy in MRI [41, 42]. Three MRI runs were averaged to attain the best possible image quality based on gray and white matter contrast signal to noise ratio, and no susceptibility artifacts. The total acquisition time was approximately 18 h for each case. Scanner sequences were modified to permit very large encoding matrices and high resolution.

### Histology processing

Temporal lobe blocks were cryoprotected in a 20% glycerol/2% dimethyl sulfoxide solution for two weeks. Tissue blocks were sectioned coronally on a freezing sliding microtome (Leica Biosystems Richmond Inc, Buffalo Grove, IL) at 50*μ*m, collected serially, and frozen at –20°C in cryoprotectant solution. During sectioning, blockface photographs were collected serially using a Canon EOS-1D Mark IV camera (Canon, Tokyo, Japan) with a compatible LED ring flash mounted above the microtome. Tissue for Nissl staining was selected at approximately every 10 sections and thus separated by 500*μ*m. Tissue sections were hand mounted onto glass slides, dried overnight, and stained with thionin for Nissl substance. For the Nissl staining protocol, slides were defatted in a chloroform and 100% ethanol solution, followed by pretreatment in an acetic acid, acetone, 100% ethanol and water (1:1:1:1) mixture, and stained in 8% buffered thionin for 5 min. Then, the slides were differentiated in 70% ethanol with 5–10 drops of glacial acetic acid to the ideal contrast before they were dehydrated in a series of ethanol solutions with increasing concentration, cleared in xylene, and finally coverslipped with Permount (Fisher Scientific, Fair Lawn, NJ).

### Immunostaining

Immunostaining was performed on free-floating sections in 12-well plates. Adjacent sections to Nissl-stained sections were selected for immunostaining. Sectioned tissue was washed three times in phosphate buffer saline (PBS) to rid it of cryoprotectant glycerol solution. Sections were incubated with 0.5% Triton X-100 in 3% hydrogen peroxide for 20 min to quench endogenous peroxide, then rinsed in PBS three times for 5 min each. The tissue was blocked using 5% nonfat dry milk in PBS for 2 h at room temperature and rinsed in PBS. All primary antibodies (Table 2) were diluted in 1.5% normal goat serum (NGS) (Jackson Immunoresearch, West Grove, PA) and incubated overnight at 4°C. The monoclonal (mouse) phosphorylated tau antibody CP13 (gift from Dr. Peter Davies) was used at 1:200 dilution in PBS with NGS [43]. CP13 recognizes phosphorylated paired helical filament tau at the serine 202 epitope and is an ideal antibody to visualize pretangles and mature NFTs [44, 45]. As an added measure, we validated our protocol by staining a subset of our cases with the antibody AT8, which recognizes similar epitopes at serine 202, serine 199, and threonine 205 [46, 47]. AT8 is the phosphorylated tau antibody commonly used for Braak staging for its ample immunoreactivity for tau proteins regardless of length of fixation time in formaldehyde [4]. Other studies have found similar staining patterns between CP13 and AT8 and have not found any significant differences in their staining due to the shared epitope at serine 202 [45, 47]. The pathology observed in our cases stained with both CP13 and AT8 was indistinguishable from each other, and for our purposes interchangeable (Supplementary Figure 4). The secondary antibodies (goat anti-mouse for CP13, AT8, and *α*-synuclein; goat anti-rabbit for TDP-43) were diluted in 1.5% NGS in PBS at 1:200 and incubated for one hour at room temperature (Jackson Immunoresearch, West Grove, PA). Tissue was subsequently washed with PBS and incubated with an ABC kit (Vector Laboratories, Burlingame, CA) for 30 min at room temperature. To visualize the staining, tissue was incubated with chromogen 3’3-diaminobenzidine (DAB kit, Vector Laboratories, Burlingame, CA) in a solution of 0.003% 3’3-diaminobenzidine and 0.3% hydrogen peroxide for 10 min. Tissue was rinsed three times with PBS prior to mounting on slides and air dried overnight. Immunostained sections were dehydrated in a series of ethanol solutions with increasing concentrations, cleared with xylene, and coverslipped with permount (Fisher Scientific, Fair Lawn, NJ). Negative control sections were processed with the identical procedure, but the primary antibody was omitted. All experiments were run with a positive control tissue section.

**Table 2:** Primary antibodies used with the name, species, and primary dilution outlined as well as source company and catalog number

Primary Antibodies	Species	Primary Dilution	Company	Catalogue #
CP13	Mouse	1:200	Gift from Dr. Peter Davies	–
AT8	Mouse	1:500	ThermoFisher Scientific	MN1020
TDP-43	Rabbit	1:500	Proteintech	10782-2-AP
*α*-synuclein	Mouse	1:500	ThermoFisher Scientific	32-8100

### Neuropathologic diagnosis and staging

Tissue sections immunostained for CP13 were assessed for AD staging and severity by three raters (MPF, JCA, JLR). CP13 stains for hyperphosphorylated tau both at the pretangle state, in which neurons display nonfibrillar punctate regions, and at the later stage in which they form intra- and extra-neuronal fibrillary tangles [4, 48–50]. It has been suggested that CP13 may be more specific to pathological tau aggregates than AT8 because the latter cross-reacts with microtubule-associated protein 2C (MAP 2C) [47, 51, 52]. To evaluate tau severity, we used Braak and Braak staging, a highly reliable method which identifies six stages of phosphorylated tau distribution across the transentorhinal and entorhinal regions (stage I and II), the neocortex of the fusiform gyri, lingual gyri, and association areas (stage III and IV), and the peristriate and striate regions (stage V and VI) [4, 5, 7, 53]. For the majority of our cases (*n* = 6), the NFTs, pretangles, and neuropil threads (NTs) were confined to the perirhinal cortex area 35a (Braak and Braak stage I) and entorhinal cortex (Braak and Braak stage II) [4, 5, 7]. A subset of our cases (*n* = 4) displayed isolated or small numbers of NFTs and/or NTs in the hippocampal subfields CA1 and CA2. These hippocampal NFTs, however, were not band-like at CA1, nor was CA2 compromised with large and strongly stained NFTs, excluding them from a stage III classification [4, 5]. All cases were evaluated for dystrophic neurites surrounding neuritic plaques in CP13 immunostained tissue; such plaques were morphologically distinct when present (Supplementary Figure 3). Five of our cases were negative for neuritic plaques, placing them in the primary age-related tauopathy (PART) category in the preclinical AD continuum [9]. All cases except for one (case 10), showed some TDP-43 immunoreactivity in the EC and dentate gyrus, giving them a classification of at least a stage 3 in the scheme by Josephs et al. [54]. Case 10 showed TDP-43 positive structures only on the amygdala and EC, giving it a stage 2 classification according to Josephs et al. [54]. We selected preclinical cases with a presence of AD-related pathology but an absence of clinical symptoms for dementia, which is optimal to investigate the early interval of cognitive decline.

### Parcellation of entorhinal cortex subfields

We parcellated the subfields of the EC in Nissl-stained sections according to Insausti et al., 1995 using a Nikon SMZ1000 microscope (Micro Video Instruments, Inc., Avon, MA). The eight entorhinal subfields parcellated were the olfactory subfield (EO), which is the most anterior and located in the medial parahippocampal gyrus, the rostral subfield (ER) located anteriorly and adjacent to EO, the lateral rostral subfield (ELr) found anteriorly near the collateral sulcus, the medial intermediate subfield (EMI) situated in the gyrus ambiens, the intermediate subfield (EI), which is positioned between the gyrus ambiens and the collateral sulcus, the lateral caudal subfield (ELc), which is an intermediate-posterior subdivision located long the posterior opening of the collateral sulcus, the caudal subfield (ECs) located posteriorly on the crown of the anterior parahippocampal gyrus, and finally the caudal limiting subfield (ECL), which is the most posterior subdivision in the entorhinal cortex, and is located most posterior on the crown of the anterior parahippocampal gyrus. We used the semiannularis sulcus as a medial landmark for EO and EMI (see Supplementary Figure 1c, d). Note the acronym EC refers to the whole cortex, and we added an “s” to the ECs that refers to the subfield (entorhinal caudal subfield). Table 3 outlines our eight anterior-posterior levels, and the respective lengths, subfields, and neighboring landmarks for each.

**Table 3:** Eight anterior-posterior levels in the medial temporal lobe with the entorhinal subfields typically present at that level, as well as surrounding anatomical landmarks characteristic of that level

AP Level	EC Subfields	Nearby Anatomical Landmarks
1	EO	anterior to the amygdala
2	EO, ER, ELr	anterior amygdala
3	ELr, EI, EMI	posterior amygdala
4	ELc, EI, EMI	genu, hippocampal head
5	ECs	pes region, gyrus uncinatus
6	ECs	dentate gyrus and hippocampal head
7	ECs, ECL	posterior-most hippocampal head, gyrus intralimbicus
8	ECL	anterior hippocampal body

AP, anterior-posterior.

### Anterior-posterior levels of the entorhinal cortex

Eight levels were established to investigate tau density in the anterior-posterior axis. We used three main criteria to establish uniform anterior-posterior levels: representation of all EC subfields, the presence of major anatomical landmarks in the surrounding hippocampal and amygdala regions, and consistent spacing between the levels themselves. The first and most important criterion defines the cytoarchitectural definition of each subfield [29]. The second criterion pertains to the fact that certain subfields reside at the same anterior-posterior level (Table 3) and represents neighboring anatomical landmarks of the adjacent structures—hippocampus and amygdala. Individual anatomical variability may change the exact appearance of the EC subfields across levels, but on average they become visible as follows: Level one is anterior to the amygdala and is composed primarily of the EO subfield. Level two is at anterior amygdala, and includes the EO, ER, and ELr subfields. Level three is at posterior amygdala and contains the ELr, EI, and EMI subfields. Level four is at the most anterior tip of the hippocampal head (genu) and includes the ELc, EI, and EMI subfields. Level five is at the pes region (elevations or digitones according Duvernoy) in the hippocampus (gyrus uncinatus) and has ECs present. Level six is where the dentate gyrus first appears in the hippocampal head and continues to have ECs present. Level seven is located at the posterior-most hippocampal head (more specifically, the gyrus intralimbicus) and the anterior most hippocampal body begins (see black arrow in Fig. 5g and Supplementary Figure 1g). This level includes the ECL subfield and sometimes ECs. Level eight is at the anterior hippocampal body and has the ECL subfield present. The third criterion outlines the range within levels (how large each level is) and ensures consistent spacing and thorough representation of the entire EC by having at least one millimeter between levels. Perirhinal cortex subdivisions consisted of Brodmann area 35a and 35b. Table 3 displays the subfield groupings, anatomical landmarks, and the anterior-posterior levels. See also Fig. 5, Supplementary Video, and Supplementary Figure 1 for visualization of the major anatomical landmarks in the surrounding hippocampal and amygdala regions at each level.

### Semi-quantitative rating scale: tau density

Densities of tau burden (NFTs, NTs, pretangles, and sometimes neuritic plaques (n = 5)) on individual EC subfields were semi-quantitatively scored using a rating scale which builds upon Braak and Braak’s well established rating scale [4, 5, 7] by focusing on the preclinical stages of dementia (Braak and Braak I and II). This semi-quantitative rating scale exclusively focuses on the earliest phosphorylated tau depositions within the anterior parahippocampal gyrus. More specifically, the scale relies on qualitative density scores within the entorhinal subfields and perirhinal cortex. The scale contained five scores, which were as follows: Score of 0 means no visible CP13-positive structures. Score 1 denotes with isolated NFTs and almost no NTs. Score 2 indicates NFTs greater in number, NFTs more densely packed than score 1 and NTs were present but densely packed. Score 3 signifies a moderate amount of densely packed, strongly stained NFTs, along with a band-like formation corresponding to EC layer II and III neurons, and NTs appear more homogeneous. Score 4 means many closely packed, darkly immunostained NFTs, numerous pretangles and CP13 immunoreactivity engulfs EC layers II and III neurons, along with a homogeneous distribution of NTs blanketing the entire area. Supplementary Figure 2 depicts examples for the semi-quantitative rating scale, illustrating the 5 possible scores. Two raters (JLR, EMW) implemented the semi-quantitative protocol to ensure inter-rater reliability; the raters used the same microscope, the same slides, and the semi-quantitative rating protocol (scores 0–4) described above.

### Quantitative tau burden in medial temporal lobe subdivisions

In addition to inter-rater tests performed to confirm reliability in the semi-quantitative protocol, we further validated our scores by employing a quantitative method that provided a supplementary reading of tau burden in these preclinical cases. The quantitative tau analysis was conducted using ImageJ/Fiji v.1.53c [55, 56] and a semiautomatic tool written in-house. Images were digitized at a 10x magnitude using a BZ-X810 Keyence Fluorescence microscope (Keyence, Osaka, Japan). The selected histopathological slides were preprocessed by manually segmenting the white matter and transferring the image to 8-bit (grayscale). The mean and standard deviation of the individual pixel gray values of the white matter were calculated (background, holes, tears, and overlaps were manually excluded). For each subfield on each slide, the tau positive area was segmented by setting an image specific threshold based on gray value (threshold = mean gray value – 0.75 ^*^ standard deviation). Finally, the number of tau positive pixels (pixel gray value under threshold) and tau negative pixels (pixel gray value over threshold) were extracted and used to calculate the tau ratio (tau ratio = number of tau positive pixels / number of tau negative pixels) as a quantitative measurement of region-specific tau burden.

### Manual labeling on ex vivo MRIs and tau semi-quantitative scores

Using cytoarchitectural features within the EC and the surrounding structures, we manually matched the coronal MRI slices with immunostained tau sections and Nissl-stained sections. Supplementary Figure 1 shows an example of the adjacent Nissl-stained sections used for immunohistochemistry in Fig. 2. The characterization of the entorhinal subfields has been demonstrated in ultra-high-resolution *ex vivo* imaging (7 T) measuring cortical thickness, volume and surface area, and validated to Nissl staining [57]. The semi-quantitative tau scores of each subfield were translated to a color code (blue, green, orange, rust, burgundy) to convey a severity map in early tau stages. Blue labeling (semi-quantitative score 0) means no visible tau/NFT burden. Green labeling (semi-quantitative score 1) means very low tau/NFT burden and essentially an isolated tangle in the region. Orange (semi-quantitative score 2) conveys a moderate tau burden, defined as NFTs greater in number and more densely packed than those seen with score 1 and discontinuous NTs. Rust (semi-quantitative score 3) translated to a high tau/NFT burden and typically showed tangles more closely packed than those with score 2, and in higher numbers; heavier NTs observed but not yet homogenous. Burgundy (semi-quantitative score 4) showed the greatest density for phosphorylated tau at CP13, with many closely packed, darkly immunostained NFTs and pretangles along with a homogeneous distribution of NTs blanketing the area. The semi-quantitative scores per subfield were manually labeled onto the corresponding MRI slides using Freeview, the FreeSurfer brain visualization program (https://surfer.nmr.mgh.harvard.edu/). This manual labeling provides the ability to reconstruct tau density in a three-dimensional volume.

### Cortical thickness measurements

Cortical thickness for each entorhinal subfield and perirhinal cortex was manually measured as the Euclidian distance from the pial surface of each subfield to the gray/white matter boundary using the measure function in Freeview. Three different slides were chosen for each subfield: when the subfield first appears in the *ex vivo* MRI scan, at the midpoint of its anterior-posterior length, and the last instance the subfield is present. Three equidistant medial-lateral locations were collected on each measured slide. Nine manual cortical thickness measurements were collected per subfield and per case, totaling 810 measurements. The multiple measurements per subfield were done to account for the changing anatomy across the medial-to-lateral and the anterior-posterior axes. Measurements for the subfield EMI were intentionally excluded due to the deformation from the test tube during the *ex vivo* MRI scans. Thickness measurements were averaged and correlated with mean CP13 semi-quantitative scores for each subfield. See Table 4 for the cortical thickness averages for each respective subfield.

**Table 4:** Average cortical thickness (manual measurements) for each EC subfield and average CP13 SQ scores for the corresponding subfield

EC Subfield	Average Cortical Thickness (mm)	Average CP13 Tau SQ Scores (0–4)
EO	4.08	1.27
ER	4.39	1.67
ELr	4.04	1.45
EI_Med_	3.24	1.73
EI_Lat_	3.31	1.95
ELc	3.10	2.18
ECs_Med_	2.80	2.70
ECs_Lat_	2.68	2.95
ECL	2.78	2.87

SQ, semi-quantitative.

### Statistics

Non-parametric correlations (Spearman’s *ρ*) were used to evaluate semi-quantitative and quantitative measurements for entorhinal burden across anterior-posterior levels. Spearman’s correlation tests were also used to determine agreement between semi-quantitative and quantitative measurements and to test the relationship between average CP13 scores and cortical thickness of each subfield. Pearson’s R was used to evaluate correlations between age and anterior pathology levels. Mann-Whitney U was used for nonparametric comparisons between semi-quantitative tau found in lateral and medial bifurcations of the EI and ECs subfields, as well as perirhinal tau. Cohen’s Kappa was used to assess inter-rater reliability on semi-quantitative tau burden scores. Kruskal Wallis and Dunn’s tests for post-hoc analyses were applied to determine statistical group differences in semi-quantitative as well as quantitative measurements across anterior-posterior levels and across subdivisions. Kruskal Wallis and Dunn’s tests were employed to assess statistical group differences in entorhinal and perirhinal cortical thickness measurements. All statistics used an alpha level of *p* < 0.05 as the threshold for significance, and were performed using Prism (GraphPad software corporation, San Diego, CA) and R-studio v.1.4.1 (The RStudio Team).

## RESULTS

### Novel entorhinal parcellation based on tau vulnerability

**Fig. 1:**
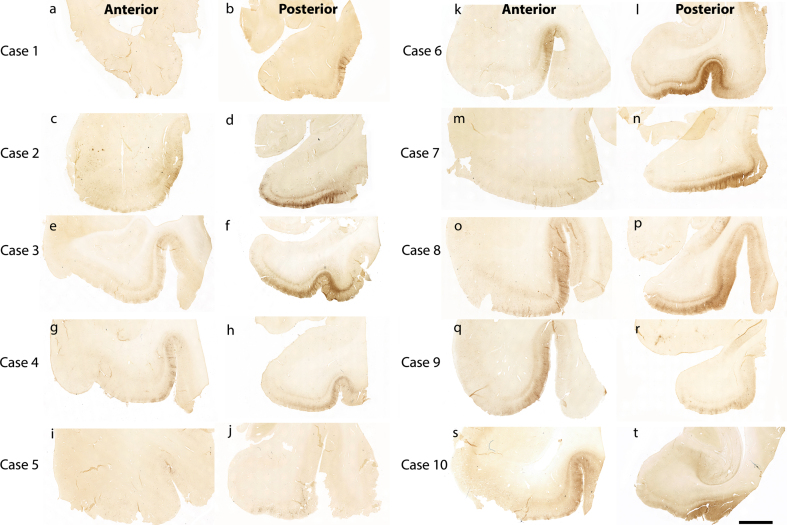
Tau immunostaining at two anterior-posterior levels in entorhinal cortex: All 10 cases illustrated with CP13 immunostained sections for phosphorylated tau early pathology. The left panels show the CP13 staining at anterior levels (a, c, e, g, i, k, m, o, q, and s) and the right panels display the staining at posterior levels (b, d, f, h, j, l, n, p, r, and t). These two anterior-posterior levels were selected to show representative differential pathology between the two. Note the high tau pathology at posterior levels and relatively low pathology at anterior levels. Magnification bar = 5 mm.

Figure 1 shows 10 preclinical (Braak and Braak I and II) cases stained and evaluated for CP13 phosphorylated tau pathology across the anterior-posterior axis (Fig. 1). Qualitatively, we found high tau density in the posterior portions (Fig. 1b, d, f, h, j, l, n, p, r, t) compared to the anterior portions (Fig. 1a, c, e, g, I, k, m, o, q, s). This pattern was observed within the entorhinal and perirhinal regions, and notably in every case.

**Fig. 2:**
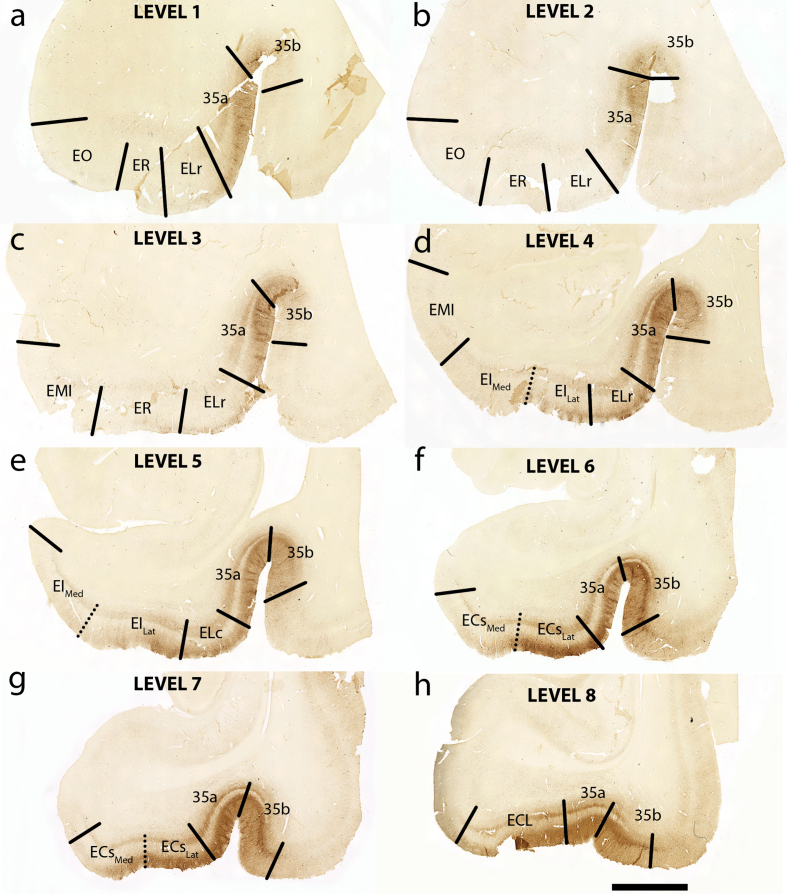
Tau Immunostaining and Novel Parcellation at Eight Anterior-Posterior Levels: Eight medial temporal lobe levels from anterior to posterior of a representative case (case 6, Braak and Braak II), immunostained with phosphorylated tau (CP13) antibody. All tissue sections illustrated in coronal plane. The following levels list a nearby landmark that best identifies each coronal level. Level One = anterior to the amygdala (a), Level Two = at anterior amygdala (b), Level Three = at posterior amygdala (c), Level Four = genu, anterior hippocampal head (d), Level Five = pes region (gyrus uncinatus) in the hippocampal head (e), Level Six = hippocampal head and dentate gyrus (f), Level Seven = posterior-most hippocampal head ends (gyrus intralimbicus) (g) (see also Fig. 5g and Supplementary Figure 1g), Level Eight = at anterior hippocampal body (h). EO, entorhinal olfactory; ER, entorhinal rostral; EMI, entorhinal medial intermediate; EI, entorhinal intermediate; ELr, entorhinal lateral rostral; ELc, entorhinal lateral caudal; ECs, entorhinal caudal; ECL, entorhinal caudal limiting; ECs_Lat_, ECs Lateral; ECs_Med_, ECs Medial; EI_Lat_, EI Lateral; EI_Med_, EI Medial. Magnification bar = 5 mm.

**Fig. 3:**
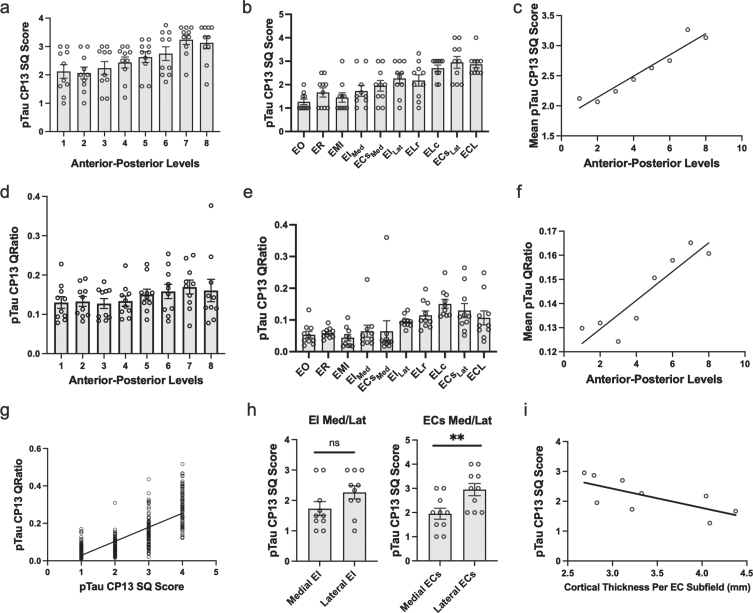
Semi-Quantitative Scores, Quantitative Measurements of Tau Burden Density and Cortical Thickness in the Entorhinal Cortex: a-c) Tau data graphed according to level or subfield. a) The mean semi-quantitative CP13 tau scores across all anterior-posterior levels within the EC. The most severe and least variable (std = 0.503) tau burden was observed at level seven. b) Mean semi-quantitative CP13 tau scores compared to entorhinal subfield. The densest CP13 pathology occurred in ELc, ECs_LAT_, and ECL. c) Positive Spearman’s correlation between anterior-posterior levels and mean semi-quantitative scores; *r* = 0.956. d-f) Quantitative tau data graphed according to level or subfield. d) Mean quantitative measurements (area-to-tau ratio) across anterior-posterior levels within the EC. e) Mean quantitative area-to-tau ratio measurements graphed by entorhinal subfield. ECs_LAT_ and ELc had the highest scores, with ELr and ECL closely tied for third highest quantitative measurement. f) Positive Spearman’s correlation between anterior-posterior level and mean quantitative tau ratio measurements; *r* = 0.90. g) Positive Spearman’s correlation between semi-quantitative scores and quantitative tau ratio measurements. h) Semi-quantitative CP13 scores for the novel bifurcations of the medial and lateral portions of entorhinal caudal subfield (ECs) and the entorhinal intermediate subfield (EI). The ECs subfield, located posteriorly, showed significant difference between medial and lateral parcellations but EI did not. i) Negative Spearman’s correlation between average EC subfield cortical thickness measures and their corresponding CP13 tau semi-quantitative scores; *r* = –0.80. EO, entorhinal olfactory; ECL, entorhinal caudal limiting; ECs, entorhinal caudal; ECs_Lat_, ECs Lateral; ECs_Med,_ ECs Medial; ER, entorhinal rostral; EMI, entorhinal medial intermediate; EI, entorhinal intermediate; EI_Lat_, EI Lateral; EI_Med_, EI Medial; ELr, entorhinal lateral rostral; ELc, entorhinal lateral caudal; pTau, phosphorylated tau; SQ, semi-quantitative.

At the preclinical stage tau pathology has not yet engulfed the entirety of the EC, but a pathology gradient is formed. We therefore segmented the boundaries of the entorhinal subfields based on phosphorylated tau staining and neuronal vulnerability to NFTs. Figure 2 shows the novel parcellation and new boundaries of the entorhinal subfields. More specifically, we bifurcated the ECs and EI subfields into lateral (ECs_Lat_ and EI_Lat_) and medial components (ECs_Med_ and EI_Med_); these subfields indicate crucial transition regions from anterior to posterior portions of the EC and accordingly illustrate the most salient pathology gradient medially to laterally. We found that the lateral regions consistently displayed heavier pathology than their medial counterparts. Figure 2f shows an example, with ECs_Lat_ displaying more substantial tau pathology than ECs_Med_. Overall, tau pathology found in the ECs_Lat_ was significantly heavier than that in the ECs_Med_ (Mann-Whitney U, *p* = 0.0137; Fig. 3h). While a similar pattern was observed between tau pathology found in EI_Lat_ and EI_Med_ the difference did not reach statistical significance (Mann-Whitney U, *p* = 0.1360; Fig. 3h). Fundamentally our tau pathology findings were used to build the new subdivisions.

### Tau density within the entorhinal cortex, most severe at level seven

**Fig. 4:**
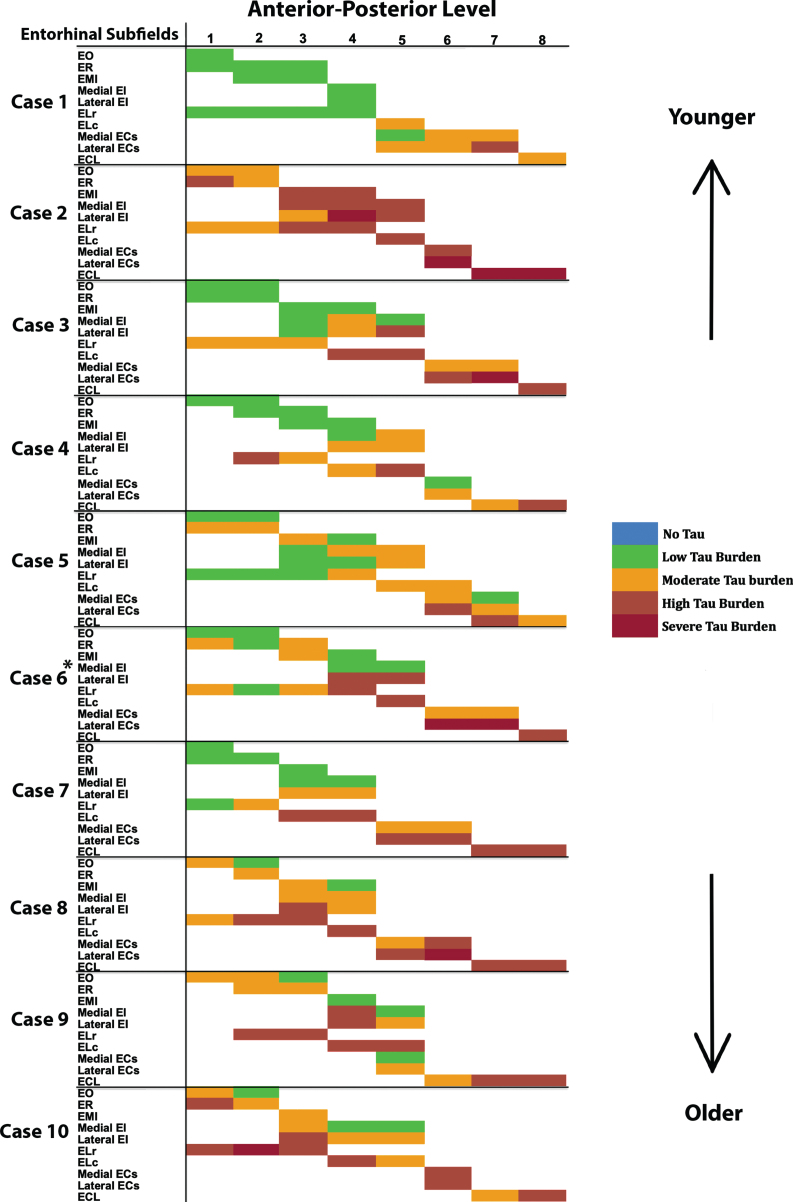
Tau Semiquantitative Scores Anterior to Posterior in EC Subfields Displayed as a Matrix (*n* = 10): Color-coded semi-quantitative tau density scores for all 10 cases showing the subfields within EC (left axis) and the anterior-posterior levels (top axis). Green labeling (score 1) means very low tau/neurofibrillary tangle burden and essentially isolated tangles in this region. Green labels were more prevalent in medial and anterior subregions. Orange (score 2) conveys a moderate tau burden both in number and packing density of NFTs. Rust (score 3) translated to a high tau burden and typically showed more densely packed and strongly stained NFTs and pretangles, with more continuous NTs. Burgundy (score 4) showed the greatest density for phosphorylated tau at CP13, with many closely packed, darkly immunostained NFTs and NTs blanketing most of the area. The position of case 6 (^*^) was predicted based on tau burden pattern. The cases have been arranged according to age, with the younger cases on the top and the older cases on the bottom (see Table 1 for specific ages for each case).

On the anterior-posterior axis, we found a strong level effect on semi-quantitative score (Kruskal Wallis H (7) = 25.56, *p* < 0.0001) and observed the most severe and least variable (std = 0.503) tau burden at level seven (Fig. 3a). Level seven is depicted as the neighboring anatomical landmark of the posterior hippocampal head (i.e., the gyrus intralimbicus). Mean semi-quantitative scores of CP13-positive structures were higher posteriorly in the EC, showing a stepwise gradient in tau density from levels one through seven, exhibited by a statistically significant positive correlation between anterior-posterior levels and semi-quantitative mean tau scores (Spearman’s *ρ*= 0.9524, *p* < 0.001; Fig. 3c). The higher pathology scores in posterior levels compared to more anterior ones is what we refer to as the “first wave” of tau pathology in EC. In this study, the “first wave” was present in all but one of the cases in the youngest-old category—those between the ages of 59 and 74 years—(Fig. 4, cases 1, 3, 4, 5, and 7; see also Fig. 6a, c, d, e, and g). Among the entorhinal subfields, we found a significant effect of subfields on semi-quantitative score (Kruskal-Wallis H (9) = 45.08, *p* < 0.01) and particularly found that three posterior-lateral entorhinal cortex subfields: ELc, ECL, and ECs_Lat_ had the heaviest tau burden density in semi-quantitative measures (Fig. 3b).

**Fig. 5:**
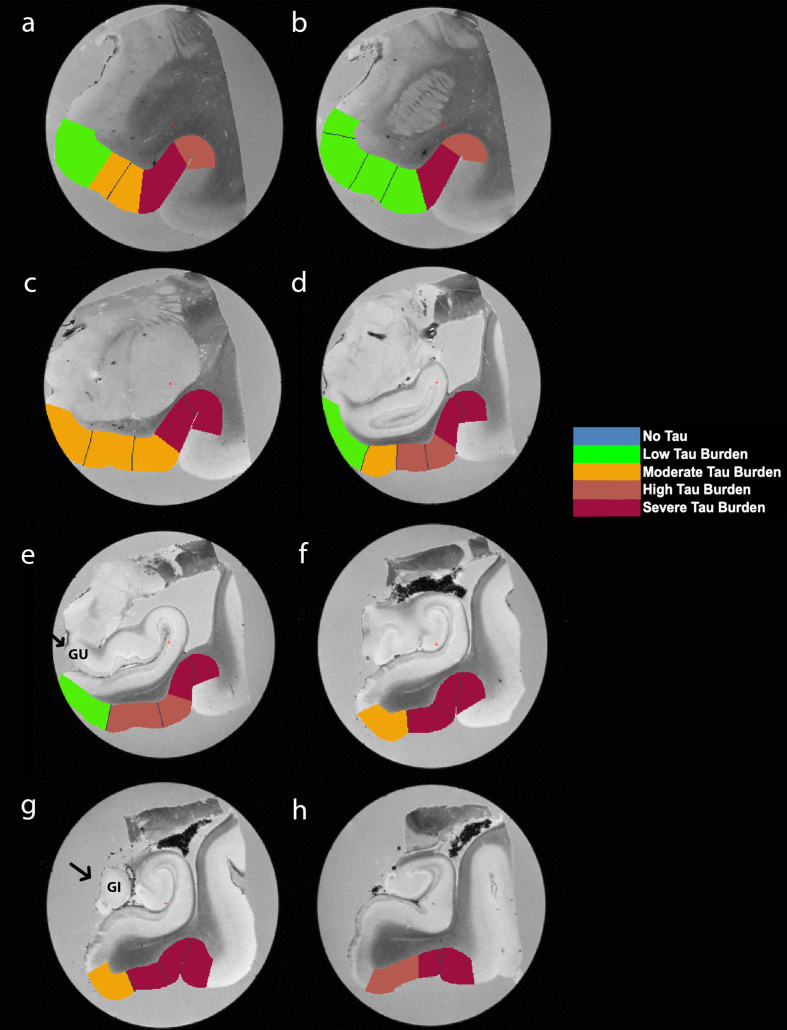
Manually Labeled *Ex Vivo* MRI: Semi-quantitative tau burden scores manually labeled on eight anterior-posterior levels (panels a-h) of a single case (case 6, Braak and Braak II). Labeled colors reflect corresponding semi-quantitative tau burden scores from Fig. 4. Blue labeling (score 0) means no CP13 tau pathology, no NFTs, no NTs. Green labeling (score 1) means very low tau/NFT burden and essentially isolated tangles in this region. Note that the green labels were restricted to medial and anterior subregions. Orange (score 2) conveys a moderate tau burden both in number of NFTs and NFT packing density. Rust (score 3) translated to a high tau burden and showed more densely packed and strongly stained NFTs and pretangles with substantial NTs. Burgundy (score 4) showed the greatest density for phosphorylated tau at CP13, with a large number of closely packed, immunostained NFTs and NTs blanketing most of the area. Black arrows point to the gyrus uncinatus (e) and the gyrus intralimbicus (g).

**Fig. 6:**
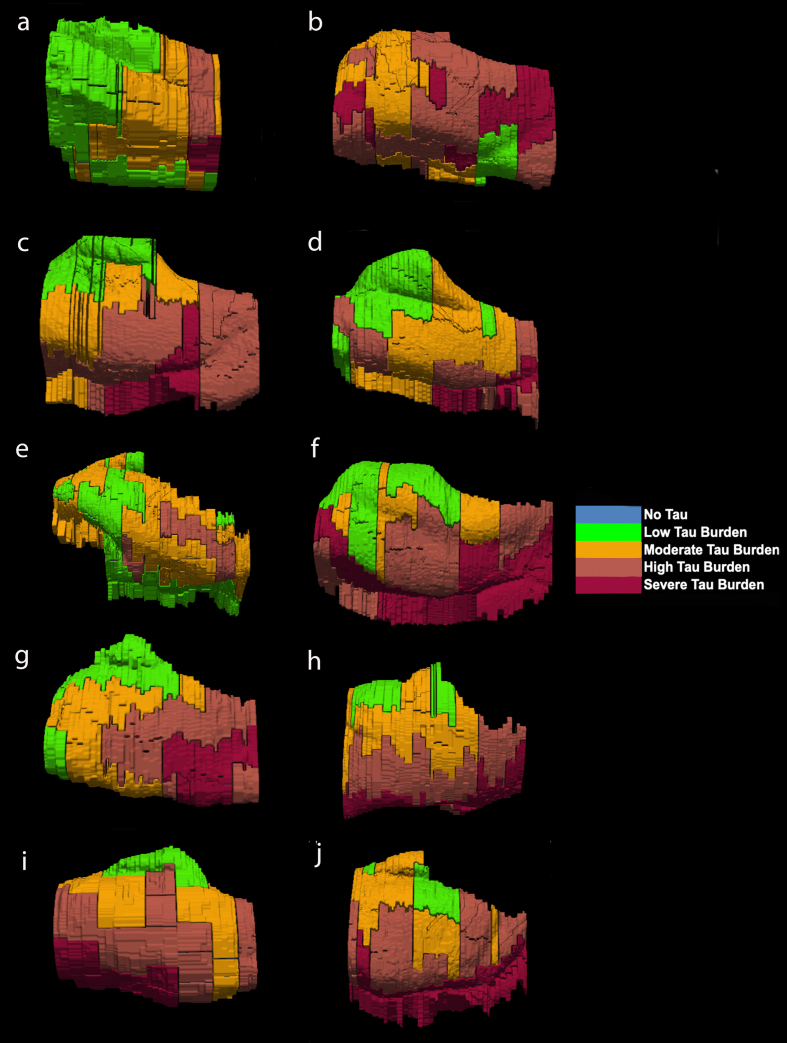
Isosurface reconstructions (*n* = 10 cases) of Manual Labeling Based on Semi-Quantitative Tau (CP13) Density Scores in Entorhinal Subfields EO, ER, Elr, EMI, EI, Elc, EC, ECL: Isosurfaces show histopathologically validated labels for the entorhinal subfields and perirhinal cortex. The labeled colors correspond to semi-quantitative scores in Fig. 4 and Fig. 5. For ease of comparison, the isosurfaces have been arranged in consistent orientation regardless of hemisphere laterality. Cases c, d, and h are blocks from right hemispheres and cases a, b, e, f, g, i, and j are blocks from left hemispheres.

For middle-old cases—those between the ages of 75 and 84 years—we observed what we deemed a “second wave” of tau pathology in the EC, which refers to high semi-quantitative scores of tau-positive structures in both anterior and posterior levels (Fig. 4, cases 8, 9, and 10; Fig. 7h, i, and j). Older age predicted higher levels of anterior pathology, as shown by a significant positive correlation observed between pathology in the most anterior entorhinal level (level 1) and age (Pearson’s *R* = 0.6867, *p* < 0.05). One case (Fig. 4 case 6; Fig. 6f) displayed the “first wave” of tau pathology but lacked demographic information and was therefore excluded from statistical tests concerning age. Given the reliability and hierarchical nature of tau pathology [58], the age of case 6 was predicted in Fig. 4 based on the presence and density of tau.

### Tau density in the perirhinal cortex

Overall, perirhinal cortex (subdivisions 35a and 35b) showed the highest tau density and was most severe at level seven (Figs. 2, 5, Supplementary Video). Mean semi-quantitative scores of CP13-positive structures were higher posteriorly in the perirhinal cortex, showing a stepwise gradient in tau density from levels one through seven. We observed a statistically significant positive correlation between anterior-posterior levels and semi-quantitative mean tau scores (Spearman’s *ρ*= 0.8264, *p* < 0.05; data not illustrated).

### Inter-rater reliability

We assessed the semi-quantitative tau pathology scores with an inter-rater reliability test. Two raters (JLR, EMW) evaluated the density of CP13-positive structures on each entorhinal and perirhinal cortices at every level in all 10 cases. Applying a Cohen’s Kappa test, we found substantial agreement among the two raters (Kappa = 0.790; 95% confidence interval from 0.740 to 0.841).

### Semi-quantitative tau scores correlate with quantitative tau measurements

In addition to inter-rater reliability, we further validated our semi-quantitative protocol with a supplementary protocol implemented to quantitatively measure tau (CP13) pathology in entorhinal subfields and perirhinal cortex. We found a strong positive correlation between semi-quantitative tau pathology scores and quantitative measurements (Spearman’s *ρ*=0.775, p < 0.01; Fig. 3g), and like the semi-quantitative scores, quantitative tau scores significantly correlated with anterior-posterior levels (Spearman’s *ρ*=0.9048, *p* < 0.01; Fig. 3f). The pattern in the quantitative tau ratio measurements was observed across anterior-posterior levels (Fig. 3d) and was similar to that observed with semi-quantitative scores (Fig. 3a). The quantitative tau ratios peaked at the level of the most posterior hippocampal head, i.e., the gyrus intralimbicus (level seven) (Supplementary Figure 1g). The entorhinal subfields with the most severe quantitative measure for CP13 pathology were found most laterally and posteriorly anatomically, with the ELc and ECs_LAT_ subfields showing the highest CP13 tau burden (Fig. 3e). ELr and ECL were closely tied for third highest quantitative measurement. The similar patterns observed between semi-quantitative scores and quantitative pathology measurements, along with the strong correlation between them, provides reliability to use the semi-quantitative scores as the foundation for the manual labeling for the *ex vivo* MRI scans.

### Manual labeling in MRI based on semi-quantitative tau scores

We manually labeled the color coded semi-quantitative tau burden density scores (0 = blue, 1 = green, 2 = orange, 3 = rust, 4 = burgundy) onto the reconciled MRI slides (Fig. 5, Supplementary Video). The same colors and scores were used in Figs. 4, 5, and 6. The MRI labels were bounded by the pial surface, the white matter boundary, and within that, we applied tau pathology parcellation obtained from the stained tissue. Together, the labels form a pathologically-validated, three-dimensional map of phosphorylated tau and NFTs in the preclinical human brain.

The reconstructed isosurfaces of the MRI labels highlight the most vulnerable segments in the tau burden severity map—the lateral/posterior portions of the EC—along with the individual anatomical variability for each case (Fig. 6). Complementing observations found in Fig. 4, the phenomenon of the “first wave” (Fig. 6a, c, d, e, g) and “second wave” (Fig. 6 h, i, j) of tau pathology in the EC was observed in the youngest-old (case 2 excluded) and middle-old cases respectively (including the one with age predicted, case 6; Fig. 6f). The labeling in Figs. 5 and 6 match the semi-quantitative scores from Fig. 4 with the anatomical level of the whole EC. More specifically, the “first wave” extends most prominently from level eight (level of the hippocampal body) to level five (level of the pes region—i.e., gyrus uncinatus—in the hippocampus) (Figs. 4 and 6). The anterior levels one (anterior to the amygdala) through four (genu of the hippocampus) were relatively spared in most youngest-old cases but are vulnerable in the middle-old group.

### Correlations between cortical thickness and CP13 semi-quantitative scores

Table 4 shows the average cortical thickness measures for each entorhinal subfield: EO = 4.08±1.04 mm, ER = 4.39±0.92 mm, ELr = 4.04±0.65 mm, EI_Med_ = 3.24±0.35 mm, EI_Lat_ = 3.31±0.23 mm, ELc = 3.10±0.31 mm, ECs_Med_ = 2.80±0.52 mm, ECs_Lat_ = 2.68±0.21 mm, ECL = 2.78±0.20 mm. Table 4 also compares EC subfield average cortical thickness and corresponding average CP13 tau semi-quantitative scores. There was a strong effect of cortical thickness on entorhinal subfields (Kruskal-Wallis H (8) = 266.7, *p* < 0.0001) and perirhinal subdivisions (Kruskal-Wallis H (5) = 16.83, *p* < 0.01), along with a significant negative correlation between average entorhinal subfield cortical thickness measures and average entorhinal subfield CP13 tau scores (Spearman’s *ρ*= –0.80, *p* < 0.05; Fig. 4i). Figures 5 and 6 show relative total area and thickness of the subfields with tau burden, with larger and thicker areas having lower tau and smaller regions showing higher tau burden. The correlation between perirhinal cortex cortical thickness (average) and their corresponding CP13 semi-quantitative scores did not reach statistical significance (Spearman’s *ρ*= –0.20, *p* = 0.71, data not illustrated).

## DISCUSSION

In summary, we investigated substructures within the EC to map the populations of cortical neurons most vulnerable to AD pathology at the preclinical stage. We found significant stepwise higher tau density from anterior to posterior levels, reaching an apex at the level of the posterior hippocampal head, i.e., the gyrus intralimbicus (level seven). Subfields ECs_Lat,_ ECL, and ELc showed the highest tau density scores across both semi-quantitative scores and quantitative measurements. We segmented the boundaries of the entorhinal subfields based on phosphorylated tau staining; the entorhinal subfields EI and ECs were bifurcated and contained heavier pathology in lateral regions compared to medial ones. In addition, we observed age-dependent anatomically specific vulnerabilities in cases with similar Braak and Braak stages. The youngest-old cases (59–74 years) displayed NFTs in posterior regions of the EC, contrasting middle-old (75–84 years) cases which showed additional CP13-tau inclusions in the anterior EC; only one case indicated exception to this pattern. Our cortical thickness measurements using *ex vivo* MRI negatively correlated with tau density scores of their corresponding entorhinal subfield, providing tissue validation to a neuroimaging measure. We mapped our CP13 semi-quantitative scores onto the corresponding *ex vivo* MRI scans (Fig. 5, Supplementary Video), and subsequently reconstructed them as three-dimensional isosurfaces (Fig. 6). The resulting anatomically comprehensive, histopathologically validated “heat maps” show subregional vulnerabilities to tau and provide a segmentation of entorhinal subfields based on early tauopathy patterns. These results narrow the focus of entorhinal NFT pathology assessment to structurally unique subfields within the EC across the rostro-caudal and medial-lateral axis in the preclinical stages of AD.

Ample evidence in the literature singles out the EC as an early location where NFTs and dementia-related cortical thinning is observed, often decades before patients become symptomatic [4–6, 8, 13, 16, 59]. Although there is little to no neuronal loss in aging and preclinical AD compared to mild AD, the predictive deposition pattern of NFTs make them reliable neurodegenerative markers to study the pre-demented brain [60, 61]. In the EC, layer II is affected first, followed by layer IV, then layer III, and finally layers V-VI [4, 5, 10]. Our semi-quantitative rating system builds upon Braak and Braak’s well established scale [4, 5, 7] by focusing on the preclinical stages of dementia (Braak and Braak I and II) and by relying on more specific (number and proximity of NFTs and NTs) density scores *within* the EC subfields and perirhinal cortex (Figs. 2, 4, 5). A quantitative protocol provided a tau ratio (number of tau positive/tau negative pixels) as a measurement of subregion-specific tau density. The semi-quantitative and quantitative protocols were applied across eight well-defined rostro-caudal levels of the MTL (Figs. 2, 4, 5) and were found to be strongly correlated with each other (Fig. 3g).

Due to the pathology gradient evident on the medial-lateral axis at these early Braak stages, we segmented the boundaries of the entorhinal subfields based on neuronal vulnerability to CP13-posititve structures (NFTs, pretangles, and neuropil threads) (Fig. 2). The lateral regions consistently displayed heavier pathology than their medial counterparts, ECs_Lat_ with significantly higher tau density than ECs_Med_ (Fig. 3h). EI_Lat_ similarly showed higher tau density than EI_Med_ but the difference was not significant. This could be related to the substantial volume and surface area variability found in the EI subfield [57]. Our results corroborate findings from Lace et al., who similarly demonstrated denser tau pathology in lateral regions of the EC in the majority of their cases [62]. Moreover, tau vulnerabilities in lateral EC regions have been demonstrated in human *in vivo* neuroimaging. Khan and colleagues measured metabolic deficits with fMRI in a human AD preclinical group and demonstrated that the lateral EC had reduced cerebral blood flow compared to controls [16]. The findings in the present study provide histopathological ground truth about the preclinical tau vulnerabilities in the lateral EC and not in medial, but what accounts for this gradient is still unclear.

Rostral, intermediate, and caudal EC subfields were described by Van Hoesen & Pandya in the rhesus monkey based on the changing cytoarchitecture from anterior to posterior regions [26, 27]. These initial parcellations were later expanded to seven subfields by Amaral et al. [63]. Insausti et al. subdivided the human EC into eight subfields based on distinct cytoarchitecture [29]. Among the EC subfields, we found ECs_LAT_, ECL, and ELc had the highest CP13 tau semi-quantitative scores (Fig. 3b). ECs_LAT_ and ELc had the highest measurements on the quantitative protocol, with ELr and ECL closely tied for third highest quantitative measurement (Fig. 3e). While the exact mechanism behind the pathological vulnerability observed in posterior EC subfields is unclear, a possible explanation may involve the molecular characterization of neurons within this region. A recent study by Leng and colleagues used single-nucleus RNA sequencing to identify the transcription factor RORB as a marker for neuronal vulnerability and susceptibility to NFTs in the caudal EC [17].

Cortical thickness of the EC is a reliable indicator of AD pathology presence and severity [64, 65]. We manually measured the cortical thickness of each entorhinal subfield and perirhinal cortex and found that semi-quantitative CP13 tau scores negatively correlated with cortical thickness of their corresponding EC subfields but not in perirhinal subdivisions (Fig. 3i). These data corroborate findings from a recent report by Ravikumar et al., which correlated atrophy in the EC with tau scores in 3D [31]. Our results extend the cortical thickness measures to the subfields, which is more sensitive and specific. While other studies have found significant cortical atrophy in BA35 in relation to tau [66], we hypothesize that because perirhinal cortex demonstrated high tau pathology even at the preclinical Braak and Braak I and II stages, we did not detect a correlation between cortical thickness and tau across the rostro-caudal axis. Atrophy in entorhinal and transentorhinal regions has been observed multiple years prior to conversion from cognitive controls to mild cognitive impairment [13], consistent with preclinical pathological changes observed in the present study. Our finding that regions with higher tau (lateral-posterior EC) display more cortical atrophy agrees with that of Kulason et al., who similarly showed higher atrophy in transentorhinal regions compared to those of the medial EC [67]. Another study by Thaker and colleagues found a significant relationship between antemortem entorhinal cortical thickness and NFTs found in the EC [8]. However, a limitation of such comparisons done by Thaker et al. is that the cortical thickness measures and neuropathologic examinations were separated by multiple years. Such comparisons with differing time intervals between MRI and death lack accurate matching of cortical thickness with neuropathology at an exact point in time. *Ex vivo* MRI has been shown to be highly related to information found in histopathological slides [68]. By validating the tauopathy and cortical thickness on the *ex vivo* MRI at the same cross-sectional stage within the EC yet over the entire extent of EC, these data represent an exact correspondence.

Despite all cases having a Braak and Braak I or II classification and no antemortem clinical symptoms of dementia, we found a unique age-dependent emergence of tauopathy in the rostrocaudal axis within our older (middle-old, 75–84 years) cases, contrasting our younger (youngest-old, 59–74 years) cases (Figs. 4, 6). Whereas all but one of our youngest-old cases had low tau (CP13) pathology density anteriorly and high tau pathology density posteriorly (the “first wave” of EC pathology), middle-old cases displayed relatively high tau density levels both posteriorly and anteriorly (the “second wave” of EC pathology) (Figs. 4, 6). Case 2 (age 59) is a notable exception to this pattern, as it shows high anterior NFT burden despite being a young case in our sample (Table 1, Figs. 1c, 4). It is well established that the greatest risk factor in AD is advanced age [69]. However, Braak and Braak I and II tau pathology has been seen in cases as early as the second and third decades of life [58]. Notably, Crary and colleagues introduced the concept of PART, or primary age-related tauopathy, to describe NFT pathology more accurately in cognitively normal aged individuals [9]. Likewise, the concept of suspected non-Alzheimer’s pathophysiology (SNAP) highlights individuals with neurodegeneration biomarkers (cerebrospinal fluid tau, MTL atrophy, and decreased metabolism in AD-like patterns) in the absence of amyloid plaque burden [70]. It should be noted that by “first wave” of pathology we are referring exclusively to cortical pathology. The locus coeruleus is the earliest location in the whole brain to show tau pathology, making it the true first “hit” of phosphorylated tau [58, 71, 72]. In the present study, we propose an asynchronous development of preclinical tau (CP13) pathology in the EC, with anterior portions developing NFTs and NTs at later stages, lagging behind the more vulnerable posterior regions. This level of subclassification would be otherwise lost if pathology is examined exclusively at a single anterior-posterior level. The extent to which coexisting pathological and neurovascular markers aggravate the progression of the asynchronous tau pathology waves described here is yet to be determined. Investigating TDP-43 further in future studies will be important, as it is present in PART cases and has been shown to strongly associate with anterior MTL regions [73, 74].

Anterograde and retrograde tracing studies in the non-human primate have elucidated crucial connectivity patterns regarding the subfields within the EC. Posterior subfields EI, ELc, ECs, and ECL receive input from the retrosplenial cortex, a spatial and episodic memory region shown to display early pathologic changes in AD progression [22, 75]. Studies in the macaque monkey have additionally demonstrated that the perirhinal cortex projects more heavily to layers I, II, and III of anterior levels of the EC (subfields EO, ER, Elr), while the parahippocampal cortex (areas TF and TH in the monkey) projects to layers I, II, III, and V of more posterior levels (subfields EI, ELc, ECs, and ECL) [21, 25]. Although it is initially counterintuitive that the region with highest cortical preclinical tau burden (perirhinal cortex) projects to the EC subfields (anterior) with lowest tau density, there are possible mechanisms that could account for this. Previous studies have shown that soluble tau aggregates (i.e., “seeds”) precede the development of tauopathy among synaptically connected structures [76, 77]. In human tissue, tau seeds have also been found in the EC with very rare NFTs and in synaptic fractions of connected non-pathology regions [76]. Based on this evidence and our findings, we speculate that the posterior pattern (“first wave”) of EC pathology (including perirhinal cortex) found in the present study develops first, depositing tau seeds on anterior subfields which develop into overt tau pathology at later stages (“second wave”). Given our sample size and the cross-sectional nature of our data, this speculation will have to be explored in the future.

Human preclinical entorhinal vulnerabilities have previously been explored via neuroimaging [13, 14, 16]. A recent study by Berron et al. used positron emission tomography (PET) imaging to investigate tau progression across MTL regions [32]. Similarly, the 2021 study by Sanchez quantified tau PET signal and found initial tau emergence in the rhinal sulcus in preclinical participants [33]. Such studies, however, lack the histopathological validation, the high-resolution correspondence achieved with *ex vivo* scans, and the well-defined and trackable anterior-posterior levels which, as shown here, have varying degrees of vulnerabilities to NFTs (Figs. 2–6). The 3D study from Yushkevich and colleagues showed vulnerability to tau among MTL regions, reporting elevated levels of NFT burden in the transentorhinal region, EC (whole), amygdala, temporopolar cortex, subiculum, and CA1 region [30]. The Yushkevich paper evaluated the entirety of the MTL, which extends posterior beyond the end of the EC, but notably, our findings corroborate their average NFT burden when considering the boundaries of EC. The Ravikumar study builds on the Yushkevich study, by combining and registering mapping to construct a 3D probabilistic atlas of MTL tau [31]. While their preliminary scores were performed on AT8-stained tissue [30], the latter slides on which the atlas was based on were immunostained with PHF-1 [31]. In our report we used CP13 (shared epitope with AT8), which is an antibody most commonly used for Braak staging for its robust immunoreactivity, lack of cross-reactivity with other tau epitopes, and reliability irrespective of fixation time in formaldehyde [4]. The Ravikumar et al. atlas similarly assesses the EC as a whole, including cortical thickness measurements, not entorhinal substructure. Here, we introduce age-specific anatomy of tauopathy distribution at the subfield level. Our work extends these initial studies by focusing exclusively on preclinical cases (Braak and Braak I and II), as well as CP13-pathology patterns in the subfields within the EC defined by Insausti et al. 1995 and bringing it into three-dimensions.

While the present study contains many strengths, it is not free from limitations. Deformation of the gyrus ambiens during the *ex vivo* MRI scans meant that the subfield EMI had to be excluded from manual cortical thickness measurements. In addition, one of our cases lacked demographic information, and had to be excluded from any analysis concerning age and its relationship to CP13 tau density. While an “n” of ten is typical for descriptive purposes, our observations regarding the age-related pathology “waves” or patterns would benefit from future studies with larger sample sizes. Our data is cross-sectional and does not provide longitudinal insight. Though these measurements individually do not represent a longitudinal approach, taken together these 10 datasets portray a perspective of tau pathology in entorhinal substructure in varying ages. Even if we cannot track pathology progression in an individual case using cross-sectional data, by arranging our cases by age (Figs. 4, 6), we can observe anterior-posterior patterns that emerge at distinct age milestones and yield pathology patterns viable for individualized medicine. Additionally, illustration of all cases (Figs. 1, 4, 6) and all levels (Figs. 2, 4, 5) in a fully disclosed and comprehensive manner showcases individual variability in anatomy, neuronal organization, and tau vulnerability. Finally, while our results provide histopathological validation of preclinical AD pathology patterns in cases with ‘typical’ Braak tau progression, atypical and early-onset AD subtypes have been identified. AD subtypes (typical, limbic, posterior, lateral temporal, MTL-sparing, minimal atrophy) present unique cognitive profiles and tau progression through differing corticolimbic networks [78–81]. Given that some of these less common subtypes are associated with early-onset AD, or present pathology that spares the MTL, it is unlikely that any of our cognitively normal cases would be categorized as atypical subtypes [78, 79]. Future studies will need to expand our EC pathology findings to include subtypes that diverge from the well-known ‘typical’ tau progression.

In summary, this descriptive study establishes tissue validation of tau staining within the entorhinal subfields by combining histopathology staining and *ex vivo* MRI. Using *ex vivo* MRI provides accurate quantitative measures that can be compared to *in vivo* neuroimaging measures, while the tau histopathology staining shows cellular vulnerability and delivers accurate pathology assessment. These detailed mappings in early tau pathology distribution may have implications in elucidating preclinical pathology propagation in this region for AD. Future imaging applications of these techniques in longitudinal studies could uncover compelling therapeutic interventions.

## Supplementary Material

Supplementary Material

Supplementary Video **Manually Labeled Ex Vivo MRI:** 3D transitioning video of semi-quantitative tau burden scores manually labeled on eight anterior-posterior levels of a single case (case 6, Braak and Braak II). Labeled colors reflect corresponding semi-quantitative tau burden scores from Fig. 4. Blue labeling (score 0) means no CP13 tau pathology, no NFTs, no NTs. Green labeling (score 1) means very low tau/NFT burden and essentially isolated tangles in this region. Note that the green labels were restricted to medial and anterior subregions. Orange (score 2) conveys a moderate tau burden both in number of NFTs and NFT packing density. Rust (score 3) translated to a high tau burden and showed more densely packed and strongly stained NFTs and pretangles with substantial NTs. Burgundy (score 4) showed the greatest density for phosphorylated tau at CP13, with a large number of closely packed, immunostained NFTs and NTs blanketing most of the area.
