# Supplementary material for: Entorhinal Subfield Vulnerability to Neurofibrillary Tangles in Aging and the Preclinical Stage of Alzheimer’s Disease
Source: J Alzheimers Dis. 2022 May 31;87(3):1379–99. doi: 10.3233/JAD-215567 (PMC9198759; doi:10.3233/JAD-215567)
Supplement: Supplementary Material [file jad-87-jad215567-s001.pdf]

Supplementary Material

Entorhinal Subfield Vulnerability to Neurofibrillary Tangles in Aging and the Preclinical Stage of Alzheimer's Disease

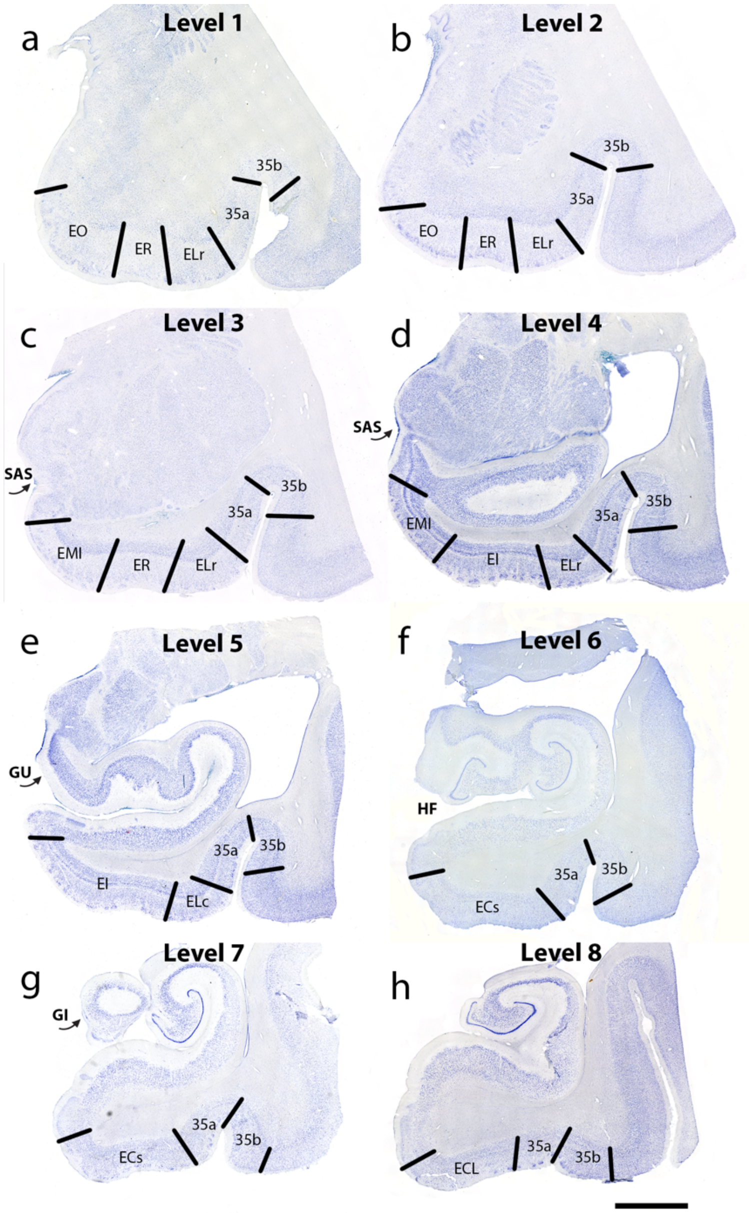

Supplementary Figure 1. Nissl Staining and Subfield Parcellation at Eight Anterior-Posterior Levels: Nissl stained (thionin) entorhinal subfields with annotations at eight anterior-posterior levels of a representative case (case 6, Braak and Braak II). This parcellation is based on Insausti's subfield parcellation (1995) and serves as a guide for the nearby immunostained tau sections. Panels a, b, c, d, e, f, g, and h represent the eight different levels in coronal plane, and each corresponds to the eight levels used to assess the phosphorylated tau in other experiments. Panel a, b, c, and d denote the more anterior levels with the amygdala shown superiorly while panels d, e, f, g designate the more posterior levels with the hippocampus shown superiorly. EO, entorhinal olfactory; ER, entorhinal rostral; EMI, entorhinal medial intermediate; EI, entorhinal intermediate; ELr, entorhinal lateral rostral; ELc, entorhinal lateral caudal; ECs, entorhinal caudal; ECL, entorhinal caudal limiting; GI, gyrus intralimbicus; GU, gyrus uncinatus; HF, hippocampal fissure; SAS, semiannularis sulcus. Magnification bar = 5 mm.

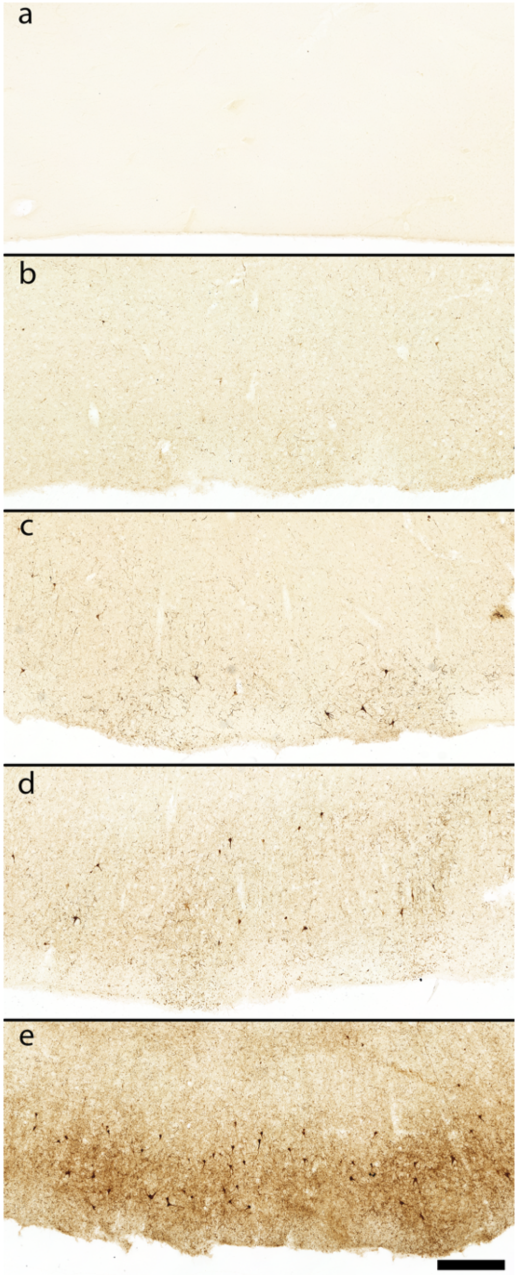

Supplementary Figure 2. The Semi-Quantitative Protocol Shows the Five Scores: CP13 tau immunohistochemistry in Photographs. CP13 semi-quantitative scores illustrated in a representative case (case 3, Braak and Braak II) to show scoring examples. The scale contained five scores: Score 0 (a) means no visible CP13-positive structures. Score 1 (b) denotes with isolated neurofibrillary tangles (NFTs) and almost no neuropil threads (NTs). Score 2 (c) indicates NFTs greater in number, NFTs more densely packed than score 1 and NTs were present but densely packed. Score 3 (d) signifies a moderate amount of densely packed, strongly stained NFTs, along with a band-like formation, corresponding to EC layer II and III neurons found and NTs appear more homogenous. Score 4 (d) means many closely packed, darkly immunostained NFTs, numerous pretangles and CP13 immunoreactivity engulfs EC layers II and III neurons, along with a homogeneous distribution of NTs blanketing the entire area. Magnification bar = 500 µm

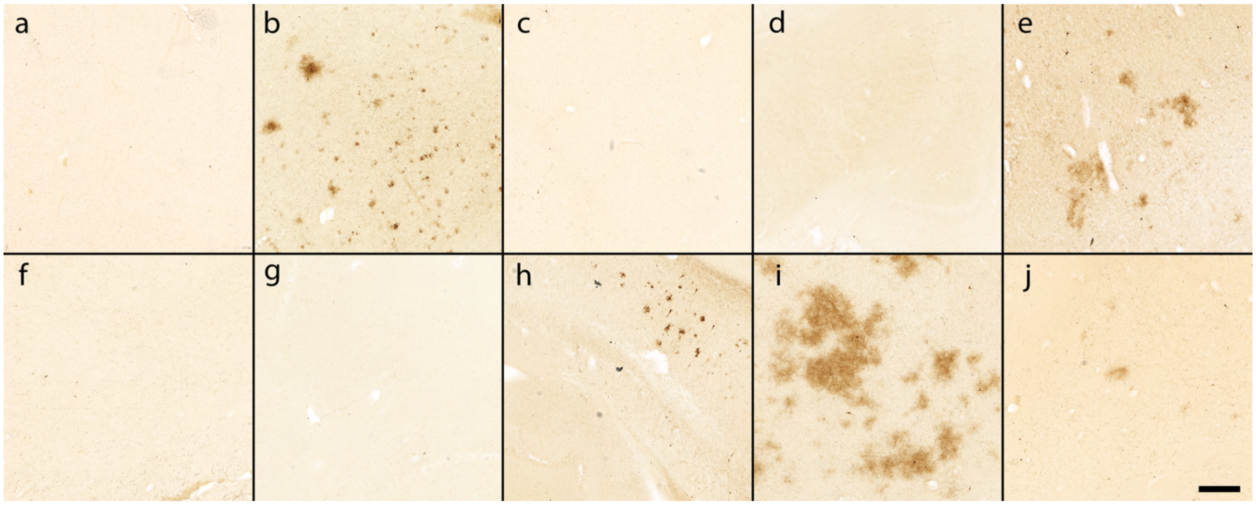

Supplementary Figure 3. Amyloid Plaque Burden in Preclinical Cases: Immunostaining shows CP13 positive amyloid plaques and mature neuritic plaques with dystrophic neurites in the medial temporal lobe. Ten cases are illustrated in the same order as other figures: panels a-j = cases 1-10, respectively. Magnification bar = 500 μm.

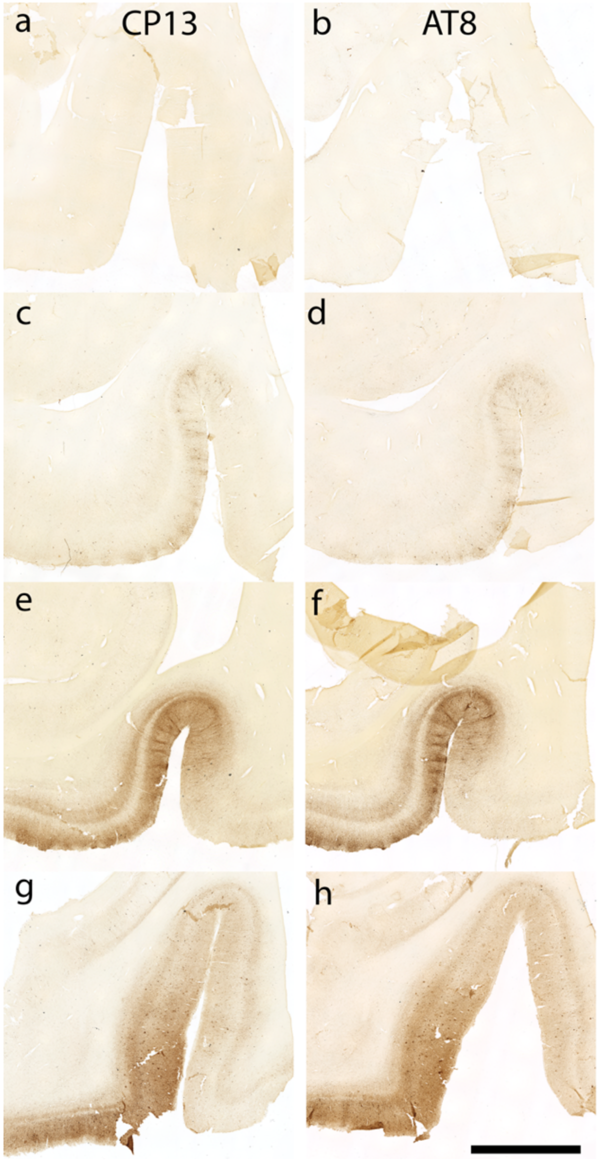

Supplementary Figure 4. Phosphorylated tau antibodies, CP13 and AT8, show similar immunoreactivity: CP13 (left column) and AT8 (right column) staining in four cases at various regional levels. Lateral-most regions of the entorhinal (area 28) and perirhinal (area 35) in a control case (a, b), a Braak and Braak I case (c, d), and two Braak and Braak II cases (e, f, g, h). All cases were immunostained with CP13 (a, c, e, g) and AT8 (b, d, f, h) primary antibodies at the same anterior-posterior level. First row (a, b) = AP level 1, AP second row (c, d) = AP level 3, third row (e, f) = AP level 5, fourth row (g, h) = AP level 8. Note the tau immunoreactivity (and thus pathology) between CP13 and AT8 appears indistinguishable from each other. Magnification Bar = 5 mm
